# Prognostic significance of focal neuroendocrine differentiation in prostate cancer: Cases with autopsy-verified cause of death

**DOI:** 10.4103/0970-1591.60442

**Published:** 2010

**Authors:** M. Tarján

**Affiliations:** Department of Pathology and Clinical Cytology, Central Hospital Falun, Sweden

**Keywords:** Adenocarcinoma, neuroendocrine differentiation, prognosis, prostate

## Abstract

**Aims:**

This study was designed to evaluate the prognostic significance of focal chromogranin A (cgA) expression in prostate cancer in a series of cases with autopsy-verified cause of death.

**Methods and Results:**

Seventy seven autopsy-verified cases of prostate cancer were identified, 41 cases with metastatic disease and 36 with nonmetastatic disease at autopsy. Immunohistochemical analysis for cgA was performed in 40 cases on the archived diagnostic biopsies taken during the patients’ lifetime. After exclusion of a single case of carcinoid tumor, 14 of the 18 (78%) metastatic and none of the 21 (0%) nonmetastatic tumors showed focal neuroendocrine differentiation (NED). The Gleason score and focal cgA expression further increased the accuracy of the prediction of the outcome, as all the cases with focal NED associated with high Gleason score had metastatic disease in contrast to cases without cgA-expression and low Gleason score, all of which were non-metastatic.

**Conclusions:**

Focal NED seems to be a powerful negative prognostic parameter in prostate adenocarcinomas. The outcome of the disease in prostate cancer can be accurately predicted based on focal NED of the tumor cells either alone or in combination with Gleason score.

## INTRODUCTION

Adenocarcinoma of the prostate is the most frequent malignant tumor in men, with an incidence of 55-65 cases per 100 000 inhabitants in the European countries. It has been estimated that the current number of 79 543 new cases per year will rise to 118 175 by 2020 in Europe.[1] On the other hand, according to the statistics of World Health Organization, prostate cancer is characterized by low cancer-specific mortality with 201000 deaths in year 2000, corresponding to 5.6% of cancer-related deaths in men or to 3.2% of all cancer-related deaths, which also means that the cause of death of most of the patients carrying this diagnosis is other diseases.[2]

Prostate cancer exhibits an obvious intertumoral heterogeneity with tumors ranging from small slowly growing lesion to rapidly metastasizing aggressive neoplasia. The clinical course and the outcome of disease remain difficult to predict. Better understanding of the biology of the disease and new reliable biomarkers are needed for adequate therapy planning to avoid over treatment or under treatment.

Introduction of the Gleason's grading system was an important step in finding valuable tools for predicting the outcome in invasive prostate carcinoma. It has become a widely accepted method by which prognostic significance and reproducibility have been repeatedly proven.[3] However, considerable inter-observer variability in grading prostate cancer has been reported in some studies, indicating a need for additional prognostic parameters.[4–6]

Several immunohistochemical panels have been tested in assessing the prognosis of prostatic adenocarcinomas, including prostate-specific antigen, prostatic acid phosphatase, CA-125, vimentin, and thyroid transcription factor as well as different cytokeratins, most often 20,7,19,8/18 and 5/6. The generated results are conflicting.[7–9] Some reports have indicated that neuroendocrine differentiation (NED) in the tumor, assessed either with immunohistochemistry or with measuring the concentracion of the product of the tumor cells in the peripherial blood, is a significant prognostic parameter associated with survival after endocrine therapy.[10–12] Basic research has shown that HER-2 (c-erbB-2) over-expression is associated with increased aggressiveness of the tumor. Slamon *et al*., was the first to report the correlation between amplification of the HER-2 gene and poor-prognosis in breast cancer in 1987.[13] Some investigators have attributed growth factor-like activity to some NE products in prostate cancer possibly influencing the HER protein family.[14]

The aim of the present study is to test the prognostic significance of chromogranin A (cgA) expression in nonmetastatic and metastatic prostate cancers as confirmed with autopsy.

## MATERIALS AND METHODS

### Study population

Seventy-seven consecutive cases of prostatic neoplasia diagnosed at the Department of Pathology, Central Hospital in Falun, between 1990 and 2007 were confirmed with autopsy completed with histological examinations. Diagnosis was made on core biopsies in 22 cases, transurethral resection specimens in 18 cases and fine needle aspiration biopsies in 37 cases. The original histological slides stained with hematoxylin and eosin were reviewed by the author. Gleason grading of the prostate carcinomas was carried out according to the official recommendations of the Urological Section of the Swedish Society of Pathology.[15]

### Immunohistochemistry

To skip the eventual modifying effect of different treatment modalities on morphology and immunophenotype of the cancer cells, immunohistochemical examination was performed only on initial biopsies in 40 cases with available transurethral resection specimens and core biopsies. Sections of 4 µm thickness from the original paraffin blocks were cut and rehydrated in graded ethanol series. Immunhistochemical staining was carried out in the Ventana ES automated Immunhistochemistry System (Ventana Medical System Inc, Tucson, Arizona, USA) using original Ventana reagents. The used primary antibody was in case of cgA (DAKO M 0869, lot. 069/201/) with 1:500 dilutions and with 32 minutes reaction time.Antigen retrieval was performed in Ventana machine with CC1 (TRIS EDTA pH 9 MILD 30 minutes). The Ventana system used biotinylated secondary goat anti-mouse antibody for the detection system and streptavidin-horseradish peroxidase conjugate for visualization of DAB solution. Endogeneous biotin activity was blocked with streptavidin.

### Statistics

Tumors were regarded as acinar adenocarcinomas with NED if single tumor cells or groups of tumor cells expressed cgA. Tumors with diffuse cgA expression were regarded as carcinoid tumor and were excluded from the statistical work up. The statistical analysis was carried out using commercially available software MedCalc (MedCalc software, Belgium), as odds ratio and Chi-square test.

## RESULTS

The mean patient age at the time of autopsy and diagnosis was 79.8 year (range 60-95 years) and 76.3 year (range 57-91 years), respectively. The average period duration between the initial diagnosis of prostate cancer and the autopsies was 40 months (range 1-150 months). Most important characteristics of the patients are illustrated in Table 1. There was no significant difference in mean time from diagnosis to death between the metastatic and nonmetastatic groups. Furthermore, the mean age of patients at the time of the diagnosis and death was almost the same in the two groups. Nonmetastatic prostate tumor could be identified at autopsy in 36 cases in which illnesses other than prostate cancer were the cause of death. In 41 cases, metastatic spread of prostate cancer was evidenced at autopsy. Most of the metastases appeared in the skeleton of the patients, especially in vertebra. Table 2 demonstrates the localization of the metastatic deposits in the present series.

**Table 1:** Characteristics of study population

	PCA with metastasis	PCA without metastasis
Mean age at the time of diagnosis in years (range)	76.7 (57-91)	76.2 (58-89)
Mean age at the time of death in years (range)	79.7 (60-94)	79.9 (64-95)
Mean time from diagnosis to death in months (range)	39.5 (1-144)	40.2 (1-150)
Mean PSA (ng/ml) at the time of the diagnosis (range)	33.1 (5,4-200)	29.8 (5-86)
Number of patients with ≥8 Gleason score	31	9
Number of patients with ≤7 Gleason score	10	27

PCA: Prostate cancer; PSA: Prostate-specific antigen

**Table 2:** Localization of metastases at autopsy in the present series of prostate cancer patients by anatomical site and by cause of death

		Causes of death	
		
Localization of metastases	All metastatic cases 41 (%)	Prostate cancer 34 cases (%)	Other diseases 7 cases (%)
Bones	29 (70)	25 (73)	4 (57)
Lymph nodes	16 (39)	13 (38)	3 (42)
Liver	15 (36)	15 (44)	-
Lung	11 (26)	11 (32)	-
Urinary bladder	6 (14)	5 (15)	1 (14)
Pancreas	4 (9)	4 (11)	-
Adrenal gland	3 (7)	3 (8)	-
Kidney	2 (5)	2 (6)	-
Stomach	1 (2)	1 (3)	-

**Figure 1:**
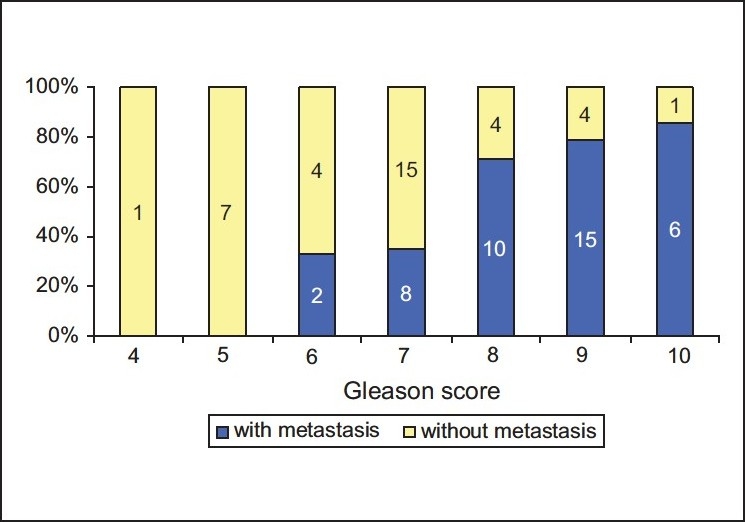
Correlation between the biopsy Gleason score and the presence or absence of metastasis in 77 cases of prostate cancer

**Figure 2:**
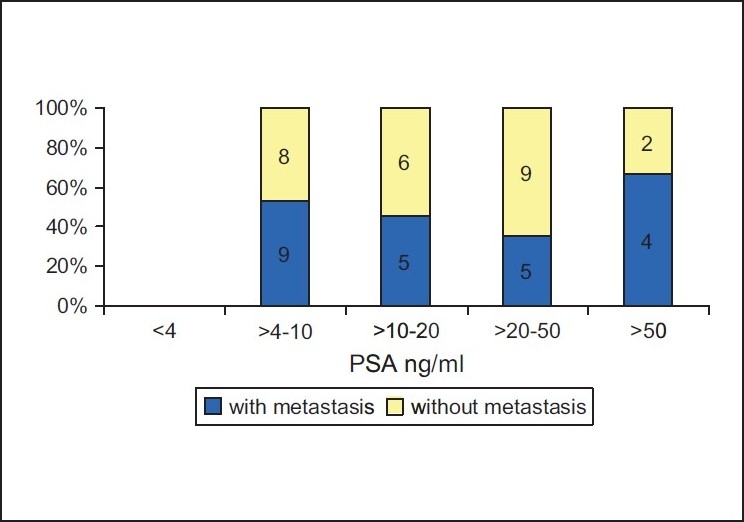
Correlation between the Prostate-specific antigen level at the time of diagnosis and the presence or absence of metastasis in 48 cases of prostate cancer

The seventy-seven cases in the present series showed the following distribution of the histological tumor types: Conventional prostate cancers in 71 cases, small cell carcinoma in 5 cases, and a single case of carcinoid tumor. It should be noted that there was no metastasis in the case of carcinoid, whereas all small cell carcinomas showed extensive metastatic disease causing death. The small cell carcinomas were not included in our further investigation. Gleason score ranged from 4 to 10 in non-metastatic cases and from 6 to 10 in metastatic cases, respectively. Figure 1 illustrates the presence or absence of metastasis in relation to Gleason score. Presence or absence of metastasis in relation to PSA data from the time of diagnosis is presented in Figure 2. There was no correlation between PSA data and metastatic prostate cancer as we can see in case of Gleason score.

**Figure 3:**
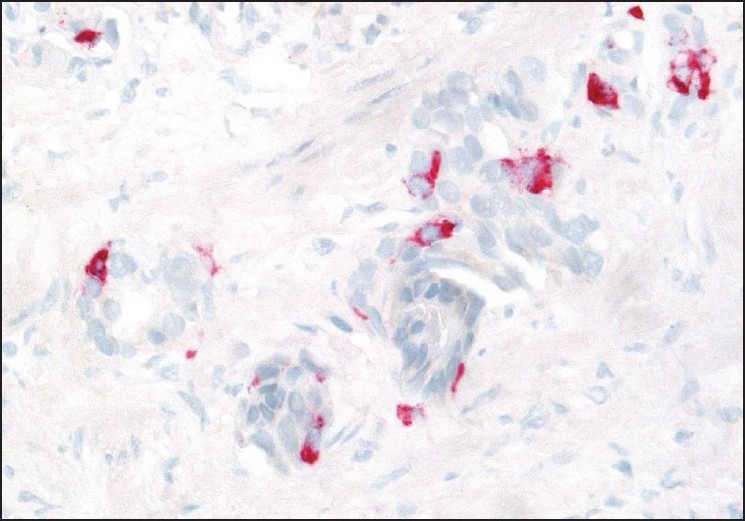
Conventional prostate cancer with focal neuroendocrine differentiation (chromogranin A staining)

According to the design of our study, cgA expression was analyzed with immunohistochemistry among the 40 cases with available archived diagnostic biopsies. We could verify focal cgA positivity in 14 cases [a typical case illustrated in Figure 3]. In addition, a single tumor showing diffuse cgA positivity was regarded as carcinoid and excluded from the study group. While focal cgA positivity appeared in 78% of metastatic tumors, all the nonmetastatic prostate cancer cases showed complete absence of cgA expression. When compared to each other, cases with focal cgA positivity showed a statistically significantly higher proportion of metastatic disease (odds ratio = 6,2500, 95% CI, Chi-square = 14,510, *P* < 0,005) than the cases without cgA expression.

**Figure 4:**
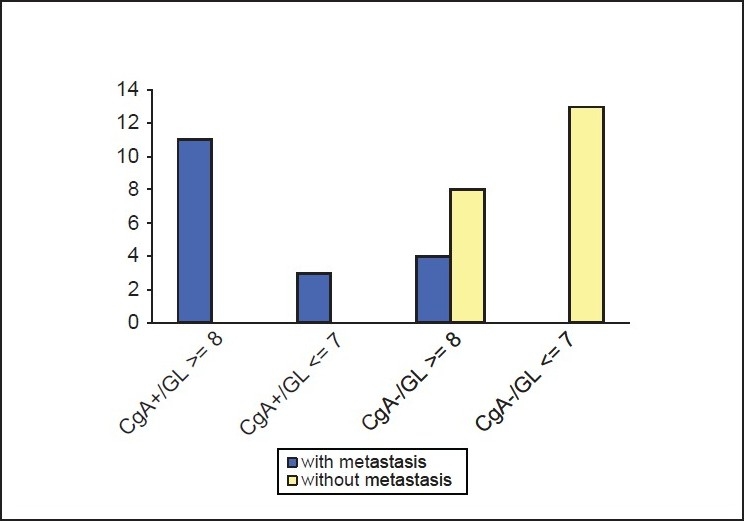
Combination of biopsy Gleason score and cgA immunohistochemical staining for the prediction of metastasis in prostate cancer (cgA: Chromogranin A, GL: Gleason score)

To enable a similar statistical approach in testing the significance of Gleason score, we grouped together our results as shown in Figure 1 as follows: Gleason score ≥8 was regarded as “high” and Gleason score ≤7 for “low” and combined these categories with the results of cgA expression. As seen in Figure 4, all the 11 cases with high Gleason score and focal cgA expression manifested metastatic disease in contrast to the 13 cases with low Gleason score and absence of cgA expression, which all were non-metastatic. The statistical analysis of the results in Figure 4 revealed high significance (Chi-square = 27,857, *P* < 0.0005), indicating that combination of these two parameters is a powerful tool in predicting the outcome of the disease in patients with prostate cancer.

## DISCUSSION

Along with secretory luminal and basal cells, neuroendocrine (NE) cells represent the third epithelial cell type in prostate glands. These basic cell types obviously share a common origin from pluripotent stem cells.[16] This concept is based on the occurrence of intermediate differentiation between these epithelial cell types.[16] Currently, two functional compartment can be defined in human prostatic epithelium; the basal cell layer represents the proliferation compartment, while the differentiation compartment consists of secretory luminal cell, which are androgen-dependent but have a limited proliferative capacity. Conversely, NE cells do not show proliferative activity and consistently lack the proliferation-associated Ki 67 (MIB-1) antigen.[17,18] These data clearly indicate that NE cells are postmitotic and represent a terminally differentiated cell population in the human prostate. Another distinct feature of prostatic NE cells is the absence of the nuclear androgen receptor (AR), thus NE cells are androgen insensitive.[19] Immunohistochemical data have shown that NE tumor cells in conventional prostate cancer express both endocrine (cgA) and exocrine (PSA) markers indicating that NE tumor cells derive from the exocrine (PSA positive) cell types and appear during tumor progression.[16]

It has been shown that prostate cancer resistant to androgen withdrawal therapies still contains AR with maintained role in proliferation of hormone-refractory prostate cancer cells. Mutations may lead to supersensitivity of AR to very low levels of androgens, growth hormones, glucocorticosteroids, growth factors or biogenic amines.[20] NE tumor cells, on the other hand, produce various peptides, hormones, cytokines and growth factors (such as serotonine, bombesin, calcitonine, vascular endothelial growth factor (VEGF), interleukine 6, interleukine 8, and cgA) that could stimulate the proliferation of surrounding non-endocrine tumor cells by autocrine, paracrine and luminecrin modulation and increase their aggressiveness through neo-angiogenic stimulation.[20,21] This concept is also supported by the results of Grobholz *et al*., who demonstrated increased Ki-67 and Polo-like kinase 1 activity in close relation to NE tumor cells.[22]

NE cancer cells do not show any proliferative activity are independent of hormonal regulation and are immortal escaping apoptosis.[23] These unusual cellular characteristics may have therapeutic implications knowing that cytotoxic agents and radiation therapy predominantly affect cycling cancer cells. In prostatic adenocarcinoma, the proliferation compartment is composed of modulated exocrine cell types while fully differentiated NE tumor cells remain in a quiescent state.[18] It is likely that nonproliferating NE cells are more resistant to chemotherapies and radiation therapy than cycling exocrine cells.

There are conflicting data reported in the literature regarding the prognostic significance of NED in prostate cancer. Many researchers have shown significant correlation between NED, tumor grade and poor prognosis demonstrating increased number of NE cells at advanced tumor stage, in high-grade versus low-grade tumors and, especially, after androgen suppression.[11,24] In contrary, other researchers did not find these correlations.[10,12]

HER-2 protein over-expression and/or gene amplification have been demonstrated in a subset of prostate cancer, especially in the androgen independent phase of disease[25] Hormonal therapy may increase HER-2 expression, as observed by some researchers.[24,26] A possible explanation is that androgen ablation therapy may induce selective overgrowth of cancer cells with high HER-2 expression.[27] As NE cells can also stimulate androgen-independent clones in the primary tumor, genetic changes resulting in phenotypically distinct non-endocrine tumor cells in their dynamic microenvironment, localized in close proximity to NE tumor cells is also possible.[14,28] Possible influence of NE cell products in prostate cancer to HER protein family is unclear. Further prospective data are needed to confirm this finding.

Tumors of the prostate with NED represent a heterogeneous group of entities, according to the new World Health Organization classification of tumors of the urinary system and male genital organs.[2] NED in the prostate cancer ranges from focal appearance of NE cells in otherwise conventional adenocarcinoma to carcinoid tumor and to small cell carcinoma. The most common histopathological pattern of malignant tumors of prostate is the conventional adenocarcinoma with or without NED.[2] There is a growing body of evidence of clinical and prognostic significance of focal NED in conventional prostate cancer, but not without divergent results in the literature. Up to our best knowledge, there are no autopsy studies, like the present one, addressing this topic. Although the number of cases analyzed in the present series is relatively low, verifying the cause of death with autopsy is a clearly advantageous limiting factor. Our approach seems to be unique in focusing on the findings in diagnostic needle and trans-urethral resections specimens retrospectively in cases with proven outcome. Our study demonstrated focal NED in biopsies in 14 of 18 metastatic conventional prostate cancer cases, in contrast to 21 non-metastatic cases all without any cgA positivity. This indicates that focal NED in initial biopsies of prostate cancer is associated with unfavorable outcome of the disease and is a simple but very powerful negative prognostic parameter. Focal NED characterizes tumors with androgen independent aggressive tumor cells clones with obvious metastatic capacity and limited sensibility to the offered therapy.[29] In such a case, alternative to the conventional hormonal treatment, for example somatostatin-analog,[14] or in advanced hormone- refractory cases combined chemotherapy with platinum- based drugs have been proposed.[30]

## CONCLUSION

Similar to several other publications, the present study underlines the importance of focal NED in prostate cancer, as evidenced in initial routine biopsies with immunohistochemical examination of cgA expression. This simple analysis enables the pathologist to provide very valuable prognostic information to clinicians in addition to other well established prognostic factors such as Gleason score, preoperative serum PSA level, number of positive biopsies, etc.[4–6] This information is also important for planning adequate therapy.
